# PomBase: The Scientific Resource for Fission Yeast

**DOI:** 10.1007/978-1-4939-7737-6_4

**Published:** 2018

**Authors:** Antonia Lock, Kim Rutherford, Midori A. Harris, Valerie Wood

**Keywords:** *Schizosaccharomyces pombe*, Fission yeast, Biological database, Model organism, Gene ontology, GO slim, Annotation extensions

## Abstract

The fission yeast *Schizosaccharomyces pombe* has become well established as a model species for studying conserved cell-level biological processes, especially the mechanics and regulation of cell division. PomBase integrates the *S. pombe* genome sequence with traditional genetic, molecular, and cell biological experimental data as well as the growing body of large datasets generated by emerging high-throughput methods. This chapter provides insight into the curation philosophy and data organization at PomBase, and provides a guide to using PomBase for infrequent visitors and anyone considering exploring *S. pombe* in their research.

## 1 Introduction

PomBase (http://www.pombase.org/), funded by the Wellcome Trust, is the model organism database (MOD) for the fission yeast *Schizosaccharomyces pombe*. Its primary goals are:

- To support the exploratory and hypothesis-driven research needs of those using the model eukaryote fission yeast as an experimental system.
- To provide an integrated model of a eukaryotic cell.
- To promote and support the use of fission yeast as a model eukaryotic system, with particular relevance to human biology.
- To provide a community hub, and support for in-depth community led curation.

To accomplish these goals, PomBase integrates the *S. pombe* genome sequence and features with genome-wide datasets and detailed, comprehensive gene-oriented manual curation of published literature, and provides tools to interrogate these data [1, 2].

As an experimental organism, fission yeast is inexpensive to grow, proliferates rapidly and is amenable to genetic manipulation. Researchers typically study isogenic strains of *S. pombe* derived from a single isolate known as 968 h90. This facilitates the comparison of results across different laboratories. Aside from the PomBase database, several organism specific resources are available to fission yeast researchers, including the genome-wide deletion mutant collection [3, 4] and the Orfeome localization collection [5].

The sequence of the reference strain 972 h–(a naturally occurring 968 h90 derivative) was published in 2002 [6]. Fission yeast has a compact genome, 14 Mb in size, that consists of three chromosomes of 3.5–5.6 Mb plus a 19 kb mitochondrial genome. 5071 protein-coding genes, of which more than two thirds are conserved in human, are annotated in the reference genome, along with rRNAs, tRNAs, snRNAs, snoRNAs, and other noncoding RNAs.

The fission yeast community comprises ~2000 researchers working primarily or exclusively with *S. pombe* or other *Schizosaccharomyces* species. In addition, fission yeast is used extensively as a complementary organism by those studying conserved cellular mechanisms in vertebrate systems, including the cell cycle, cytokinesis, chromosome segregation, epigenetic mechanisms, DNA metabolism, and drug responses [7–10]. PomBase thus serves a large (~15,000 unique users per month) and varied user base with diverse experience and requirements.

## 2 Data Curation in PomBase

The most precise and reliable molecular data in PomBase are generated by manual curation of the fission yeast literature. Automated methods, such as annotation transfer based on sequence orthology, and high-throughput datasets supplement the body of manually curated data.

To enable the fission yeast community to contribute directly to PomBase, we have developed Canto [11], an intuitive web-based literature curation tool. Canto allows both professional curators and community researchers to use state-of-the-art annotation techniques to build complex connections among genes, ontology terms, and supporting metadata. Notably, the use of ontology terms and “annotation extensions” described below underlies the generation of comprehensive curated networks representing biological processes. By combining the topic-specific expertise of biological experts with PomBase curators’ familiarity with ontologies and annotation practices, Canto usage yields literature curation of a particularly high standard of accuracy and specificity [12]. To date (August 2017) approximately 10,000 annotations have been submitted by community curators for over 500 publications.

## 3 PomBase Gene Page Organization

**Fig. 1:**
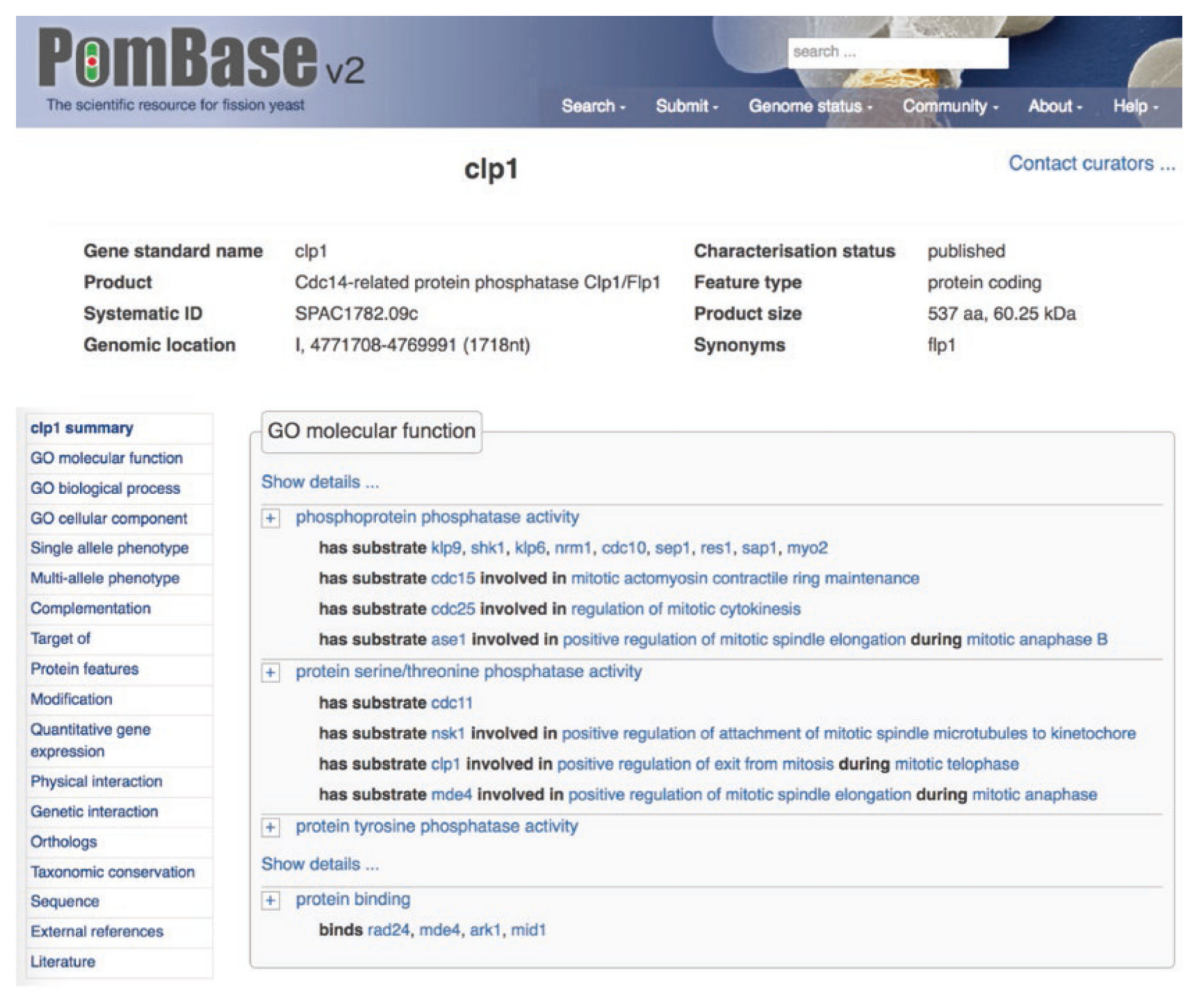
The quick-links menu. The menu displayed on the left-hand side of gene pages provides an overview of the different data types available for specific genes, and enables rapid navigation between the different sections of the gene page

Like other model organism databases, PomBase organizes data into pages summarizing genes, publications, ontology terms, and others, of which the most intensively used are gene pages. Each gene page gathers all data relevant to the gene into one place, with a menu that shows available data types at a glance and facilitates navigation within the page (Fig. 1). Gene pages can be accessed directly by typing a gene name in the search field at the top right corner of each PomBase page, and selecting it from the drop-down list (e.g., *clp1*, *cdc2*, *cdc25*, *mde4*).

Curated data types include ontology-based annotations for gene function (Gene Ontology; GO), phenotypes, and modifications, genetic and physical interactions, qualitative and quantitative gene expression data, protein features, complementation, orthologs and taxonomic conservation. Gene pages also provide gene and protein sequences, and links to gene-specific entries in external databases, and a collection of literature relevant to each gene. We discuss several of these in depth below.

### 3.1 Gene Ontology Data

**Fig. 2:**
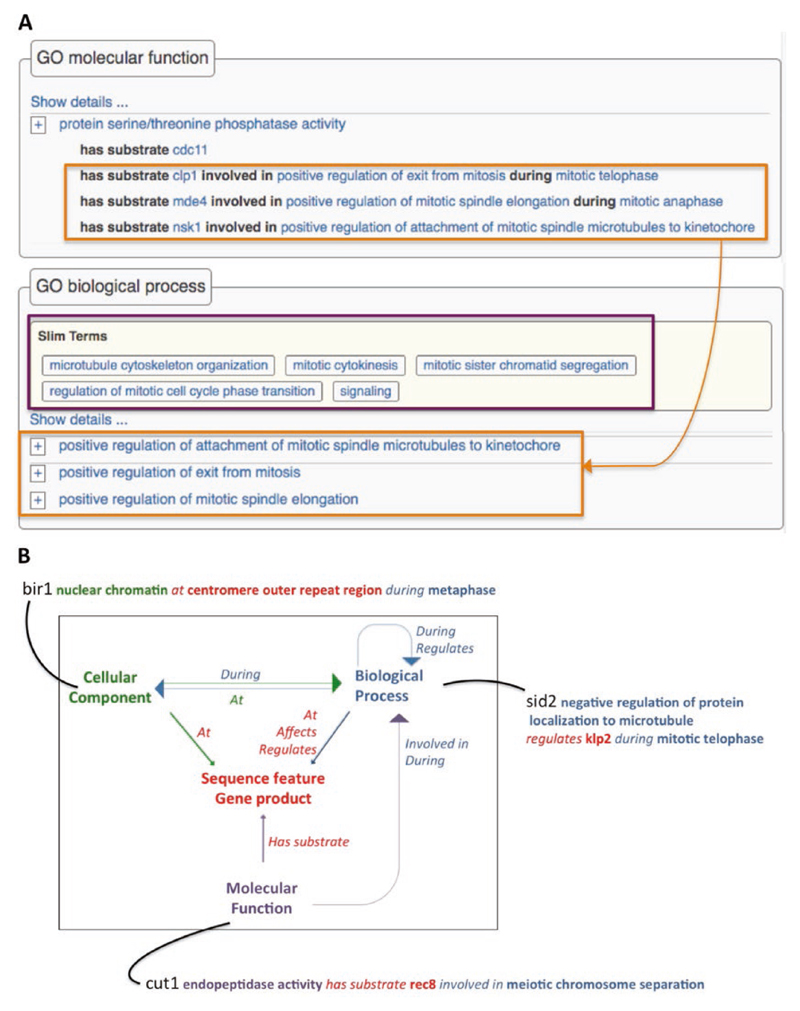
GO annotations and extensions. (A) Summary view of selected annotations on the *clp1* gene page. The orange boxes highlight annotations representing Clp1’s roles: Clp1 dephosphorylates the Nsk1 protein to positively regulate spindle attachment to the kinetochore. During anaphase, it dephosphorylates Mde4 to positively regulate spindle elongation. Clp1 also directly targets itself during telophase to promote mitotic exit. Processes linked to molecular functions are also shown in the biological process section. Biological process annotations that map to the PomBase GO slim are shown at the top of the biological process section Fig. 2 (continued) (purple box). (B) Relations used in GO annotation extensions, showing how each links one gene to other genes or additional ontology terms, with examples for each GO branch

The Gene Ontology (GO) section of the gene pages shows a table of annotations using each of the main branches of GO: molecular function, biological process, and cellular component [13, 14]. By default, the tables display a nonredundant summary of annotated terms and extensions. Figure 2A shows a selection of molecular function and biological process annotations for the protein phosphatase *clp1*. An expanded view shows all annotations as well as supporting metadata such as references, evidence, term IDs, and qualifiers. Ontology terms, genes, and references in the annotation views link to additional PomBase pages. The biological process section also lists any GO slim terms (*see* below) applicable to the gene.

The *clp1* GO molecular function annotation shown in Fig. 2A also illustrates the usage of GO annotation extensions. PomBase was a pioneer in the implementation of annotation extensions [15], which allow curation of effector–target relationships (such as protein kinase substrates) or spatiotemporal information (such as where and when a function is executed). Extensions on the *clp1* “serine/threonine phosphatase activity” molecular function annotation indicate that Clp1 dephosphorylates different substrates to contribute to different regulatory processes (e.g., Clp1 dephosphorylates Mde4 to positively regulate spindle elongation during anaphase).

Figure 2B shows a summary of the relationships used at PomBase to curate annotation extensions, and then, as described below, to build networks using the resulting connections among gene products.

### 3.2 Phenotype Data

Phenotypes are curated by PomBase using the Fission Yeast Phenotype Ontology (FYPO), an ontology of over 6000 precomposed phenotype terms [16]. Fission yeast researchers typically study isogenic strains, making it possible to define “normal” and “abnormal” phenotypes in mutants compared to the behavior of the “wild type” reference strain.

**Fig. 3:**
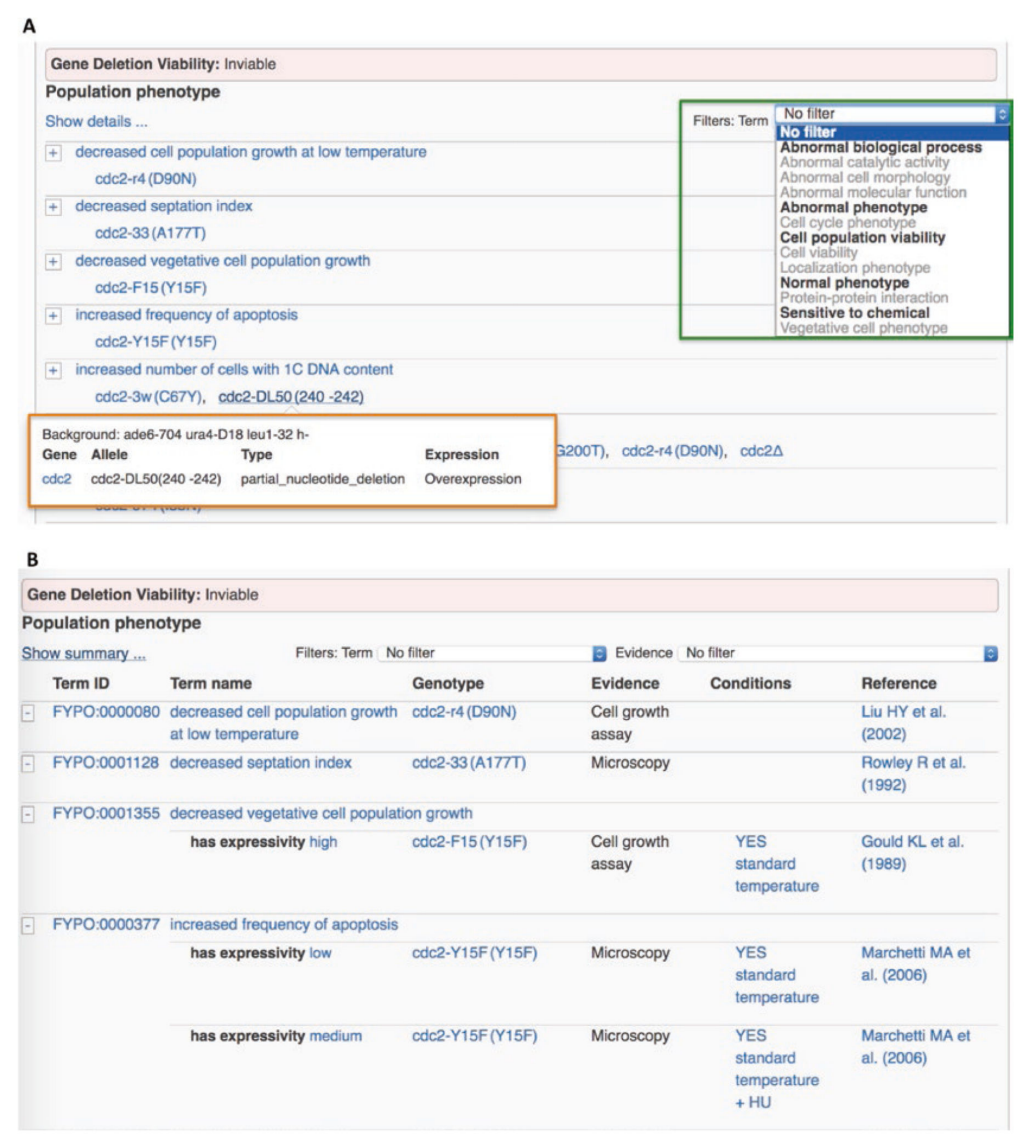
The PomBase phenotype display. (A) Summary view (B) Detailed view of a subset of phenotypes associated with alleles of *cdc2*. In the summary view, redundant annotations (including annotation to the same phenotype term, but with different extensions or metadata) and metadata are hidden. The detailed view shows all annotations, plus the cited references, evidence, extensions indicating penetrance, expressivity, or affected gene products, and any curated experimental, conditions. Genotype details, including the type of mutation for each allele, expression level of the gene products, and any background genotype information, are provided in a mouse-over (shown in A, orange box). A drop-down menu enables filtering for subsets of phenotypes (shown in A, green box)

PomBase curates single mutant allele and multiallele genotypes, which are displayed in separate gene page sections. The phenotype view is further split into population and cell level phenotypes and users can toggle between a summary view (Fig. 3A) and a detailed view (Fig. 3B). Gene deletion viability is indicated at the top of the single mutant phenotype section. The displayed phenotypes can be filtered by broad phenotypic categories (viability, cell cycle, morphology, etc.), improving the usability of the very long phenotype lists now present for many genes (green box, Fig. 3A).

**Fig. 4:**
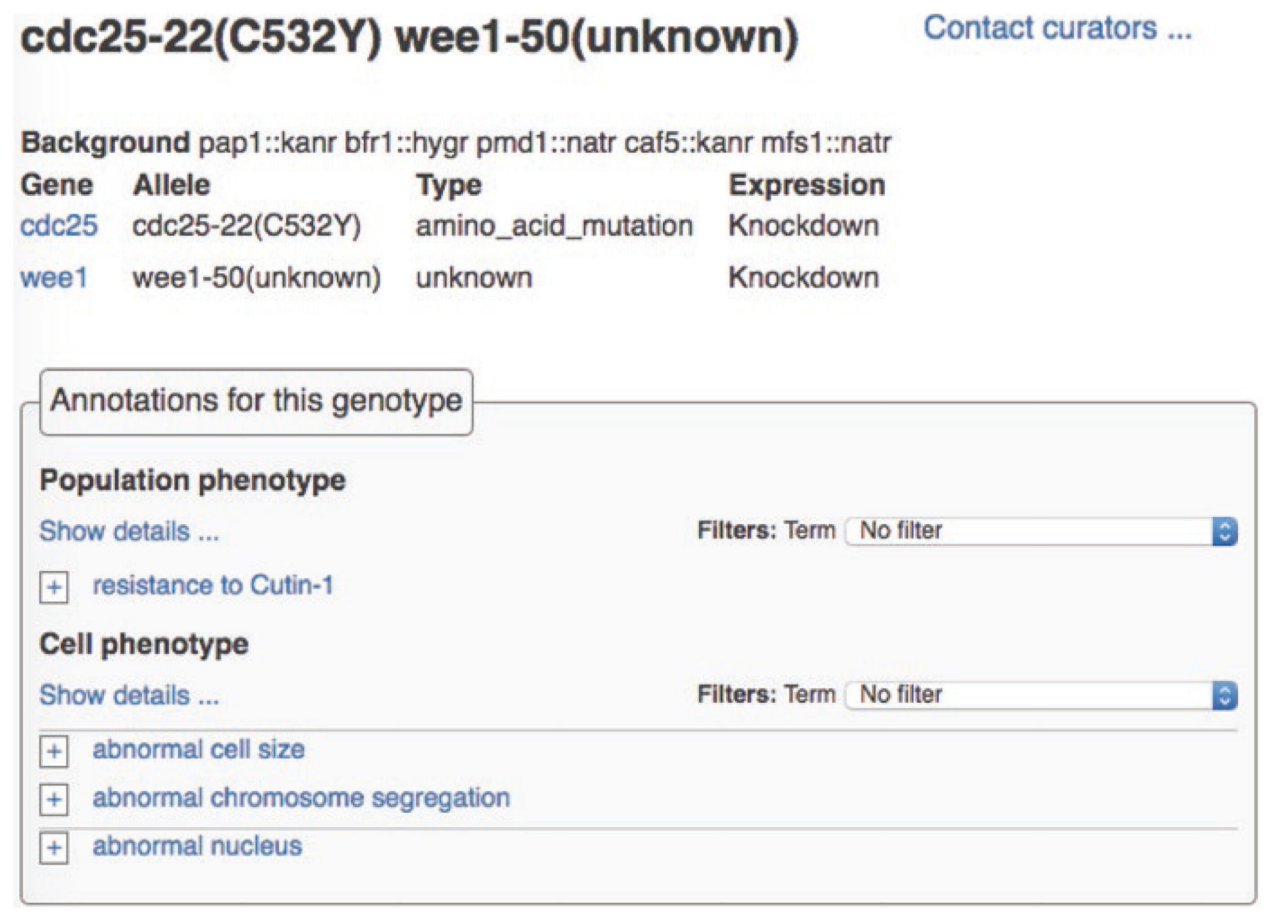
Genotype pages. Each genotype page displays allele and expression details and all annotations associated with the genotype. In this example, the double mutant comprising *cdc25-22* (C532Y) and *wee1-50,* both at reduced expression levels, in the background *pap1::kanr bfr1::hygr pmd1::natr caf5::kanr mfs1::natr* has been associated with four different phenotype terms

Each phenotype annotation also links to a page dedicated to the genotype, which displays details (name, description, expression level) for the alleles that make up the genotype, any background alleles, and all annotated phenotypes (Fig. 4).

### 3.3 Targets

**Fig. 5:**
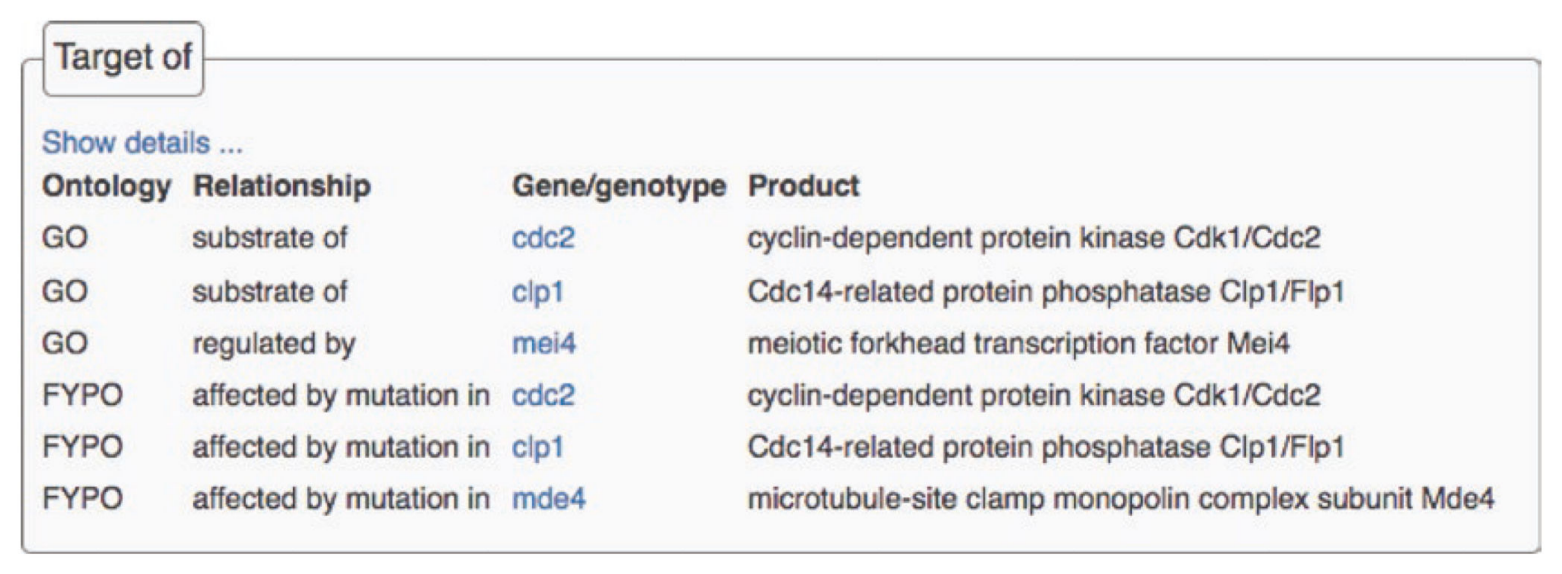
The *mde4* “Target of” section. Because *cdc2* is annotated to a protein kinase molecular function term, with Mde4 specified as a substrate, *cdc2* is listed in the “target of” section for *mde4*. Reciprocal annotations are also generated from phenotype and protein modification annotations. For example, a mutation in *cdc2* has an effect on *mde4*, with phenotypic details available on the *cdc2* gene page, and a “target of” annotation using the “affected by mutation in” relationship on the *mde4* gene page

**Fig. 6:**
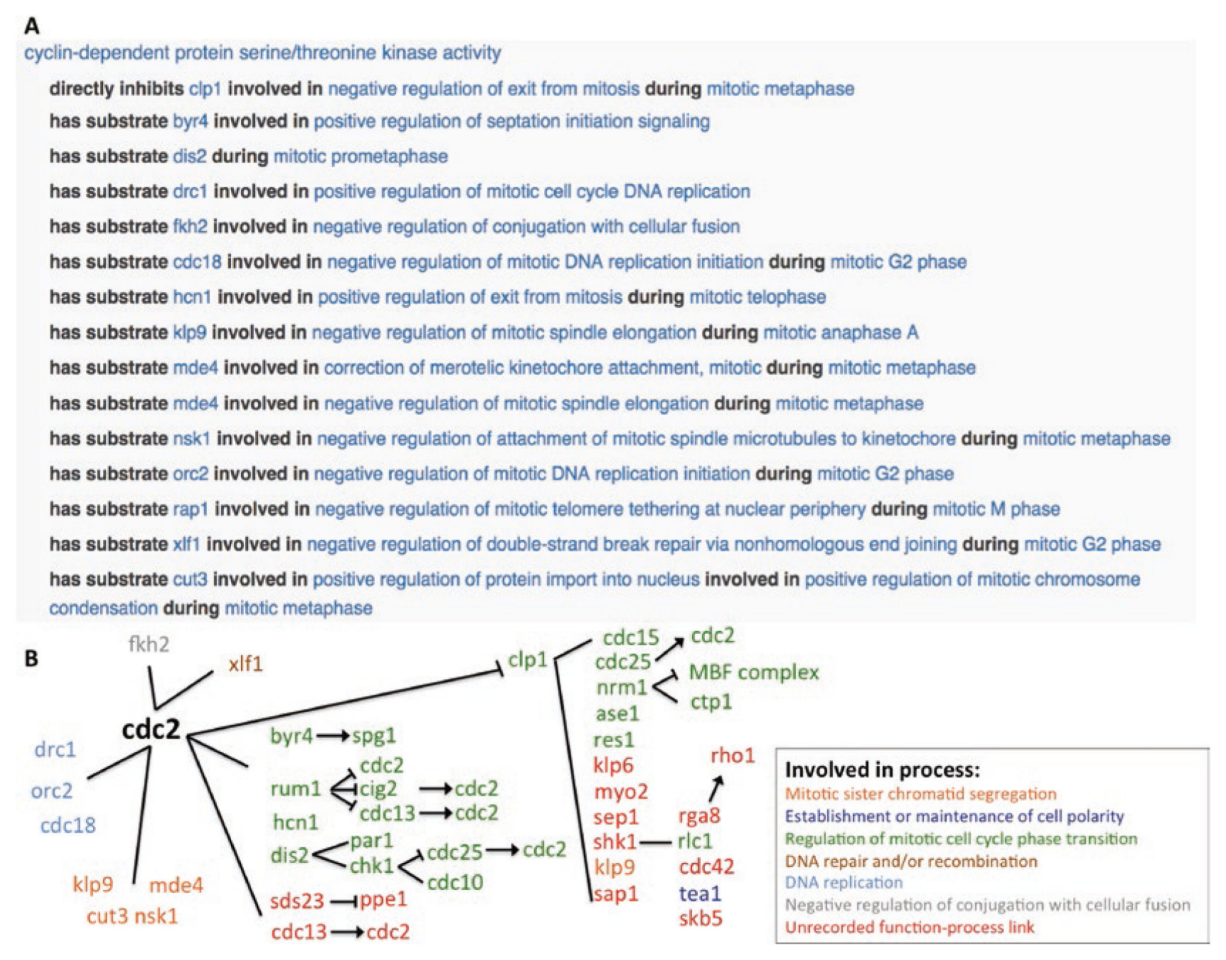
*cdc2* function–process links and downstream signaling cascades. (A) Showing the subset of Cdc2 phosphorylation targets with function–process links. Biological processes that the enzyme–substrate interaction is part of, or happens during, are indicated using the “*involved in*” and “*during*” annotation extension relationships. (B) Targets downstream of Cdc2 can be accessed via the hyperlinked annotation extension substrates, enabling users to follow biological pathways. The capturing of targets makes it possible to reconstruct pathways for a systems level representation of gene networks

The “Target of” section provides information about gene products or mutations that affect the gene of interest, derived from the reciprocal of annotations specifying targeted genes, such as the substrates of molecular functions. Target annotations indicate the connecting ontology term and the specific relationship between the two genes. For example, Clp1 dephosphorylates (Fig. 2A) and Cdc2 phosphorylates (Fig. 6) the Mde4 protein. Since Mde4 is targeted by these proteins, *clp1* and *cdc2* are listed in the *mde4* “Target of” section (Fig. 5). Users can thus navigate entire biological pathways; downstream by a gene product’s GO molecular function substrates, and upstream by effectors in the “target of” section. Reciprocal annotations are also generated from phenotype and protein modification annotations.

### 3.4 Taxonomic Conservation, Orthologs, and Disease Curation

To support the growing cohort of researchers using both fission yeast and other species, PomBase maintains manually curated inventories of orthologous proteins for fission yeast vs. human and fission yeast vs. budding yeast (*Saccharomyces cerevisiae*). Both are compiled by integrating published data and in-house analyses with the consensus from numerous orthology resources [17]. The human–fission yeast curated orthology dataset now identifies human orthologs for 69% of the fission yeast proteome.

Gene pages show any manually curated orthologous genes in human and budding yeast, and the basic gene search will retrieve available *S. pombe* orthologs using human standard gene names or budding yeast systematic (ORF) names. Where a fission yeast gene has a human ortholog that has been implicated in a disease, the PomBase gene page notes the disease and a source publication.

The taxonomic conservation section shows a broad domain kingdom or phylum level conservation for protein-coding genes. Taxon restrictions are also recorded where applicable. Other terms may also be assigned, such as whether the gene is conserved one-to-one. Classifiers are assigned manually from a small in-house controlled vocabulary (Table 1).

Taxonomic conservation can be used to retrieve high quality broad taxon classification specific datasets for analyses, or to provide functional clues for unstudied proteins based on presence or absence in particular kingdom or phyla.

## 4 Building Networks

The growing body of GO annotations with annotation extensions in PomBase creates connections between gene products, and provides rich biological context to their interactions. These connections can be exploited to reconstruct biological pathways. For example, the highly conserved cyclin-dependent serine/threonine kinase Cdc2 (homolog of the mammalian *CDK1*) is known to directly phosphorylate over 140 different proteins. A number of these *cdc2*–substrate connections are linked to the biological processes that the interaction is regulating (Fig. 6A). Annotated substrates can be followed, in order to move down biological pathways (Fig. 6B).

PomBase will use the connections curated between gene products (enzyme–substrate links, and high confidence physical interaction data), and the links to the biological processes they are involved in, to automatically construct networks for biological processes. This approach will ultimately create a detailed and reliable curation-based network for a eukaryotic cell.

## 5 GO Slim Summary

PomBase maintains the fission yeast GO slim, a set of broad, high level GO biological process terms providing coverage for most gene products with process annotations (http://www.pombase.org/browse-curation/fission-yeast-go-slim-terms). Like other GO slim sets (*see* http://geneontology.org/page/go-slim-and-subset-guide), the fission yeast GO slim supports genome-level overview of GO annotation coverage, and can be used to summarize large-scale experimental results.

The PomBase GO slim terms encompass 99.5% of all genes with a biological process annotation. Uninformative (very high level grouping terms) are excluded from the PomBase GO-slim set. Table 2 shows the number of gene products annotated to each fission yeast GO slim term. Of the 5071 *S. pombe* proteins, 748 do not have a biological process annotation because their biological role is currently unknown in any species (i.e., neither the *S. pombe* protein nor any ortholog has been experimentally characterized in detail).

PomBase also maintains a list of “priority unstudied genes” for genes conserved across taxa, but not yet characterized in any species (http://www.pombase.org/status/priority-unstudied-genes).

## 6 Ontology Term Views

**Fig. 7:**
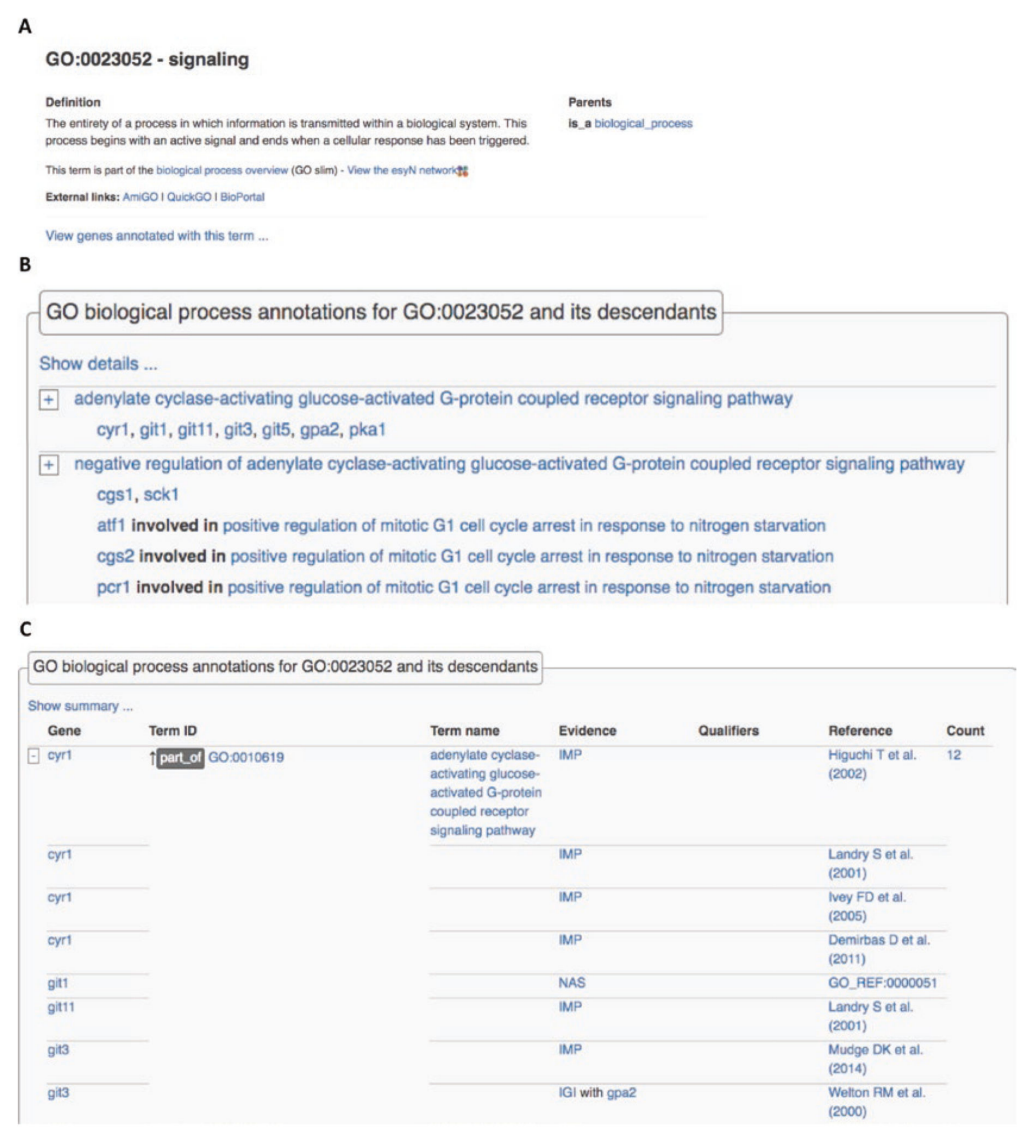
Ontology term pages. (A) The top of the page shows the term name, ID, and definition, along with immediate parent terms. Links to external resources are provided. (B) The summary view shows genes annotated directly to the term or to any of its child terms, and includes extensions. (C) The detailed view provides additional information such as the relationship of child terms to the parent term, evidence codes and references

Each ontology term used in annotations or extensions (GO, Fission Yeast Phenotype Ontology (FYPO), the Sequence Ontology (SO) [18], the chemical ontology ChEBI [19], and the PSI-MOD protein modification ontology [20]) has a term page in PomBase. The top of the term page shows the name, ID, direct links to more general “parent” terms in the ontology, and external links to relevant resources (Fig. 7A). For ontologies used directly in annotations (GO, FYPO, PSI-MOD), genes are associated with the most specific annotated descendant term (Fig. 7B shows a subset of the genes annotated directly to GO:0023052 “signaling” or any of its descendant terms). As on gene pages, the default summary view can be expanded to display annotation extensions, the type of relationship between child and parent terms (e.g., *is_a*, *part_of* or *regulates*), and supporting metadata (Fig. 7C).

## 7 Publication Pages

**Fig. 8:**
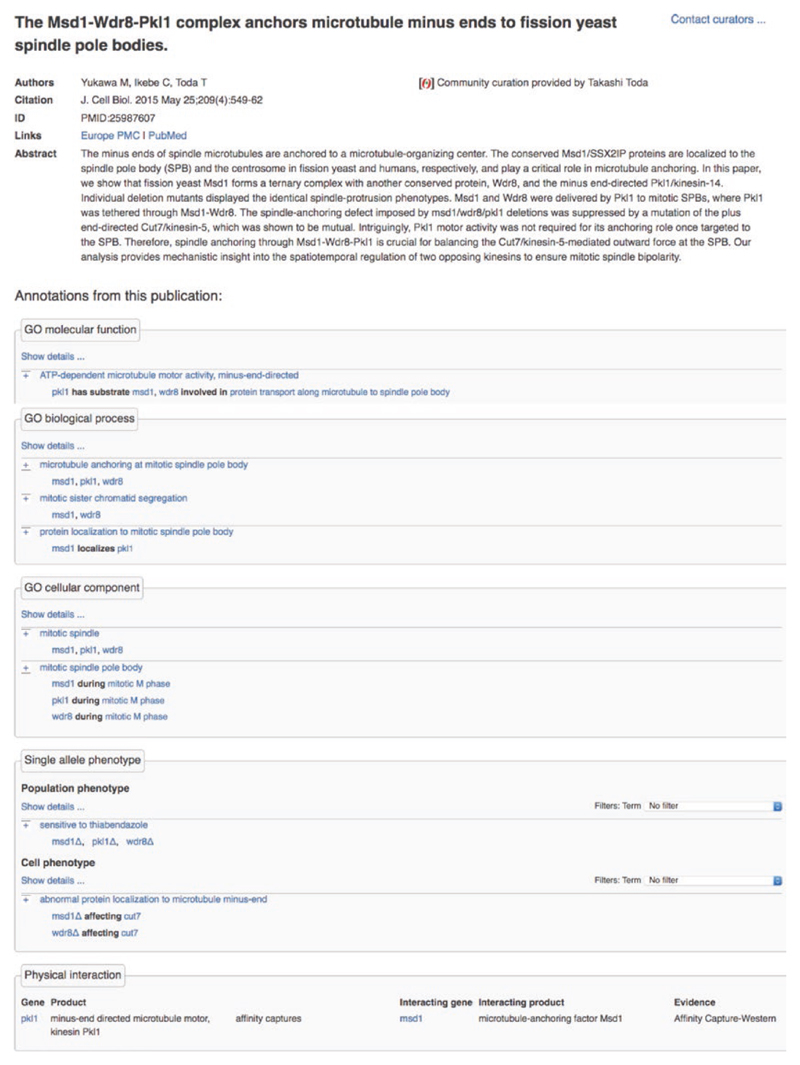
Publication pages. The PMID:25987607 page shows publication details and a community curator acknowledgement at the top, and annotations derived from the paper. GO and FYPO annotations have summary and detailed views as on gene and ontology term pages

Every paper cited in support of PomBase annotations has a publication page that displays citation details, the abstract, and all annotations curated from the publication (Fig. 8). Publication pages are connected from annotation tables and the literature section on gene pages, and from all pages that display the corresponding annotations. The page also acknowledges any community member who has contributed to the annotations derived from the publication.

## 8 Querying

PomBase offers simple and advanced search tools for querying genes and their annotations. The simple search, available on every page, retrieves individual genes by standard name, systematic ID or an *S. cerevisiae* or human ortholog name.

**Fig. 9:**
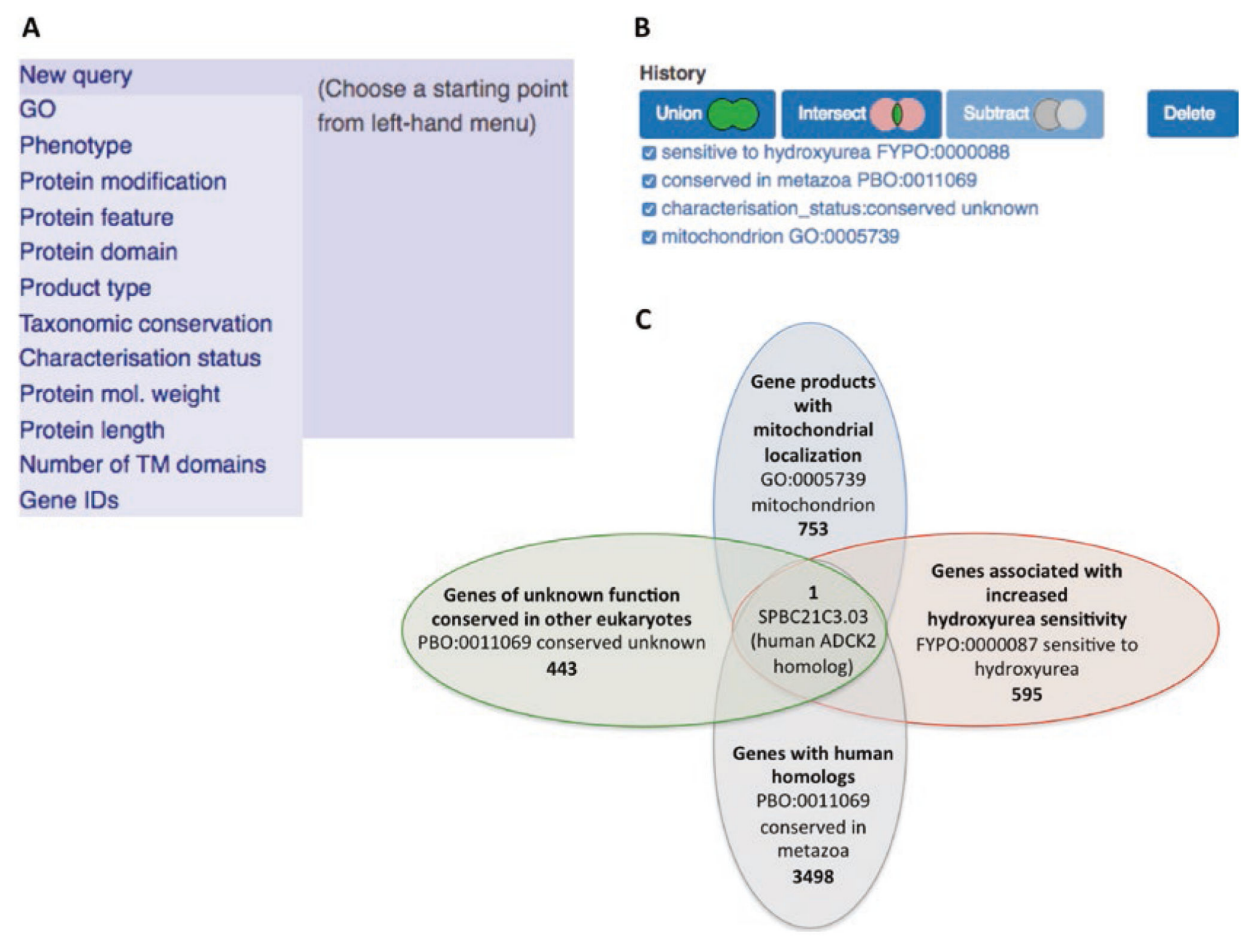
Query builder filtering. (A) A list of the different filters available to identify genes of interest. (B) The history section can be used to review previously run queries. Queries can be combined using the union, intersect and subtract operators. (C) An example of the results of running and combining queries. 753 genes (August 2017) are annotated to the GO term mitochondrion. Of these, 3498 are conserved in metazoa, 595 genes where any type of allele has been associated with hydroxyuruea sensitivity and 411 genes with the characterization status “conserved unknown,” i.e., of unknown function but conserved in other organisms. The union of these four queries identifies one gene

The advanced search retrieves sets of genes that match criteria specified by an assortment of filters (Fig. 9A). For example, ontology terms can be queried by name or ID to find annotated genes. All genes can be queried by criteria such as name, ID, product description, or chromosomal location. Additional filters are available for features of protein-coding genes. Queries can be combined to narrow down results to genes matching several criteria (Fig. 9B). Queries can be combined using the Boolean operators AND (intersect), NOT (subtract), and OR (union), and saved for reuse (Fig. 9C). For genes matching a query, IDs, names, product descriptions, and sequences can be downloaded. More flexible download options for query results are slated for addition to the advanced search.

An additional stand-alone motif search tool searches all protein coding sequences to identify genes containing a specified amino acid pattern of interest.

## 9 Genome Browser and Datasets

The PomBase genome browser supports sequence viewing based on coordinates or feature identifiers. Data tracks are available for sequence-based datasets submitted by the fission yeast community from a variety of high-throughput experiments, including transcriptomic data [21–23], nucleosome positioning [23], replication profiling [24], polyadenylation sites [25, 26], and chromatin binding [27]. (Note: at the time of writing, PomBase is in the process of transitioning from a legacy browser to a JBrowse [28] implementation.)

PomBase also provides a set of static pages describing various aspects of genome-level curation status and links to external community resources. The genome sequence and several annotation datasets (GO, phenotype, and modification data, orthologs, interactions, protein features, etc.) can be downloaded from PomBase’s FTP site.

## 10 Community and Outreach

PomBase makes community engagement a high priority, welcoming data submissions and feedback on the resources we provide.

In addition to using Canto community curation as the primary mechanism for data collection from newly published papers, PomBase invites researchers to submit large-scale datasets for phenotype, expression, and other annotations in spreadsheet-compatible formats as well as datasets suitable to appear on genome browser tracks. The most recent community curation submissions are showcased on the PomBase front page, and PomBase is exploring mechanisms for curation attribution via ORCIDs (https://orcid.org/).

We communicate with fission yeast researchers directly via our 1200-member community mailing list (pombelist) and at workshops and conferences, notably the biennial international *S. pombe* conference. To support PomBase usage, we run a helpdesk and maintain extensive documentation covering PomBase pages, annotation conventions, and Canto features. Advice on data usage and analysis disseminated via the helpdesk becomes incorporated into the extensive FAQ. Documentation and FAQs are available at http://www.pombase.org/help. We run periodic surveys to determine community priorities for new PomBase features and improvements to existing resources, and actively encourage corrections, improvements and suggestions to existing content of PomBase at all times.

## Figures and Tables

**Table 1:** Taxonomic conservation groups. Taxonomic conservation groups are assigned manually from a controlled set of terms at the kingdom/domain level. A gene may be annotated to multiple different orthologous groups. Taxon restrictions are recorded for where orthologs have not been identified outside of the noted taxa (fungi or eukaryotes). The absence of an ortholog in the *S. cerevisiae* reference sequence is also recorded. Copy number conservation is also documented, for example whether the gene is conserved one-to-one or whether orthologs cannot be distinguished. In some cases, faster evolving duplicates may be observed—this is where a copy of a gene appears to evolve faster than another copy of the gene

Orthologous groups	Conserved in archaea Conserved in bacteria Conserved in eukaryotes Conserved in fungi Conserved in metazoa Conserved in vertebrates *Schizosaccharomyces* specific *Schizosaccharomyces pombe* specific
Taxon restrictions	Conserved in fungi only Conserved in eukaryotes only
Others	No apparent *S.cerevisiae* ortholog Predominantly single copy (one-to-one) Orthologs cannot be distinguished Faster evolving duplicate

**Table 2:** Fission yeast GO slim annotations. For each term in the fission yeast GO slim, the number of annotated genes is shown. Note that a gene may be annotated to more than one slim term

GO slim term	Number of genes
Actin cytoskeleton organization	89
Apoptotic process	8
Ascospore formation	74
Autophagy	49
Carbohydrate derivative metabolic process	276
Carbohydrate metabolic process	138
Cell adhesion	20
Cell wall organization or biogenesis	104
Cellular amino acid metabolic process	190
Chromatin organization	278
Cofactor metabolic process	177
Conjugation with cellular fusion	100
Cytoplasmic translation	485
Detoxification	59
DNA recombination	122
DNA repair	177
DNA replication	118
Establishment or maintenance of cell polarity	74
Generation of precursor metabolites and energy	81
Lipid metabolic process	232
Meiotic nuclear division	112
Membrane organization	174
Microtubule cytoskeleton organization	75
Mitochondrial gene expression	167
Mitochondrion organization	146
Mitotic cytokinesis	100
Mitotic sister chromatid segregation	176
mRNA metabolic process	271
Nitrogen cycle metabolic process	16
Nucleobase-containing small molecule metabolic process	191
Nucleocytoplasmic transport	108
Peroxisome organization	22
Polyphosphate metabolic process	2
Protein catabolic process	212
Protein complex assembly	126
Protein folding	84
Protein glycosylation	68
Protein maturation	60
Protein modification by small protein conjugation or removal	98
Protein targeting	103
Regulation of mitotic cell cycle phase transition	165
Regulation of transcription, DNA-templated	415
Ribosome biogenesis	348
Signaling	292
snoRNA metabolic process	33
snRNA metabolic process	19
Sulfur compound metabolic process	109
Telomere organization	45
Transcription, DNA-templated	470
Transmembrane transport	355
tRNA metabolic process	170
Vesicle-mediated transport	329
Vitamin metabolic process	42
*Proteins with a biological process annotation not covered by the slim*	* 27*
*Proteins with no slim or biological process annotation*	*748*
